# Robot faces elicit responses intermediate to human faces and objects at face-sensitive ERP components

**DOI:** 10.1038/s41598-021-97527-6

**Published:** 2021-09-09

**Authors:** Allie R. Geiger, Benjamin Balas

**Affiliations:** Department of Psychology, North Dakota State University, Fargo, ND 58102 USA

**Keywords:** Psychology, Human behaviour

## Abstract

Face recognition is supported by selective neural mechanisms that are sensitive to various aspects of facial appearance. These include event-related potential (ERP) components like the P100 and the N170 which exhibit different patterns of selectivity for various aspects of facial appearance. Examining the boundary between faces and non-faces using these responses is one way to develop a more robust understanding of the representation of faces in extrastriate cortex and determine what critical properties an image must possess to be considered face-like. Robot faces are a particularly interesting stimulus class to examine because they can differ markedly from human faces in terms of shape, surface properties, and the configuration of facial features, but are also interpreted as social agents in a range of settings. In the current study, we thus chose to investigate how ERP responses to robot faces may differ from the response to human faces and non-face objects. In two experiments, we examined how the P100 and N170 responded to human faces, robot faces, and non-face objects (clocks). In Experiment 1, we found that robot faces elicit intermediate responses from face-sensitive components relative to non-face objects (clocks) and both real human faces and artificial human faces (computer-generated faces and dolls). These results suggest that while human-like inanimate faces (CG faces and dolls) are processed much like real faces, robot faces are dissimilar enough to human faces to be processed differently. In Experiment 2 we found that the face inversion effect was only partly evident in robot faces. We conclude that robot faces are an intermediate stimulus class that offers insight into the perceptual and cognitive factors that affect how social agents are identified and categorized.

## Introduction

Face recognition is supported by neural mechanisms that are selective for different aspects of face structure. For example, face-sensitive neural loci that have been identified by fMRI studies include the occipital face area (OFA)^1^, the fusiform face area (FFA)^2^ and the superior temporal sulcus (STS)^3^, which are part of an extended “face network” in extrastriate visual cortex^4^. These different cortical regions each have different profiles of sensitivity and selectivity that suggest different functional roles in processing face stimuli. The occipital face area appears to implement some form of part-based processing that contributes to local feature analysis within a face pattern^5^. By contrast, the FFA appears to be tuned to larger-scale, perhaps “holistic,” aspects of facial structure^6^. Finally, the STS exhibits sensitivity to multiple socially-relevant aspects of face appearance^7^. Analogous results from electroencephalogram (EEG) and event-related potential (ERP) studies of face recognition further support these functional divisions. The P100 ERP component, for example, shares some of the part-based selectivity of the OFA^8^, though it also exhibits effects of face inversion more commonly associated with holistic face processing^9,10^. The N170 ERP component, certainly the most widely studied face-sensitive ERP component, exhibits a profile of sensitivity and selectivity that suggests it functions more or less as a face detector that relies on global face appearance. For example, isolated face parts give rise to weaker N170 responses than full face patterns in some circumstances^11^, though the N170 also exhibits sensitivity to the appearance of the eyes in particular^12,13^. Refining our understanding of the image properties and image categories that elicit stronger or weaker responses from these various face-specific neural loci is an important step towards characterizing neural pathways for face recognition more completely.

An important property of face-sensitive neural responses to consider is their generality: If ERP components like the P100 and N170 are particularly large for face images, how broad is the class of face images that will elicit a strong response? By presenting stimuli that possess or lack critical visual features (e.g. specific spatial frequencies) or that belong to varying categories (e.g. animal faces) we may define the scope of face processing with regard to a specific neural response. By way of example, several studies have examined aspects of pareidolia, which refers to the perception of faces in patterns that do not truly depict faces (e.g. faces in the clouds). Pareidolic stimuli are objectively not faces, but tend to share critically important features with face patterns^14^, leading observers to label non-faces as faces under some circumstances^15^, dwell longer on pareidolic non-faces than other non-face patterns, and make websites with amusing pictures of home appliances that look like they have a personality (see the Faces in Things account (@FacesPics) on Twitter). In terms of neural responses, pareidolic faces elicit more activity from the FFA as a function of how face-like they appear to naive observers^16^. Non-face patterns that triggered false alarms from a computational model of face detection (a sort of AI-defined pareidolia) also modulate N170 and FFA responses depending on how face-like they appear^17,18^. Besides simply providing an interesting and whimsical stimulus category, these investigations of pareidolic faces offer a useful look at the boundary between faces and non-faces in extrastriate cortex. By measuring neural responses to these stimuli that are not quite faces, but not quite not faces, specific links between image features and neural responses have been established that suggest properties of the underlying representations maintained at different stages of face processing. For example, Paras and Webster^15^ found that while placing symmetric eye spots in 1/f “totem pole” images elicited pareidolia, this was not typically sufficient to elicit a robust N170 response. This kind of demonstration of what does and does not allow an image to cross the boundary from face to non-face offers useful information about the nature of face tuning at specific stages of processing.

In the current study, we chose to investigate how robot faces are processed by the visual system, which we suggest are a particularly interesting example of a stimulus category that has an unclear status in terms of how ”face-like” it may be to the visual system. Robot faces differ from human faces in terms of surface, shape, and material properties, but are also frequently designed to approximate the typical configuration of the human face. Though the appearance of robot faces differs from humans, their facial organization and pose can affect human–robot interactions^19,20^, much like human expressions. Robots’ resemblance to human faces is also sufficient for infants to expect adults to speak with robots in their environment^21^, though they do not exhibit such expectations regarding other objects. The degree of resemblance between human and robot faces is of critical importance. However, robot face likability decreases as robot appearance approaches human-like appearance, only rebounding when robot appearance becomes nearly human^22^, a phenomenon that is related to the “Uncanny Valley.” Robot face displays that look humanoid are also rated as more likely to have a mind and to be alive than robot faces that have metallic appearance^20^. Together, these results suggest that robot faces have the potential to be endorsed as social agents, though the strength of this endorsement may depend on a range of perceptual factors that lead robot faces to be either included or excluded from categorization as a face. Overall, we suggest that this unique convergence of social and perceptual factors make robot faces an intriguing category of images to examine in the context of face-sensitive neural responses. In particular, the use of ERP to characterize the visual system’s response to these stimuli makes it possible for us to investigate how robot faces are processed at specific stages of visual processing. Compared to purely behavioral measures, EEG/ERP thus allows us to examine how the tuning of face processing may differ across early and later stages of face recognition.

Our present goals were two-fold: (1) To compare the N170 response elicited by robot faces to human faces and an unambiguous non-face category, (2) To compare the N170 face inversion effect (FIE) across human face, robot face, and non-face categories. In both cases, we use the response properties of the N170 as a way to measure how face-like the neural response to robot faces is relative to human faces and objects. In Experiment 1, we used the amplitude and latency of the N170 as proxies for face-ness in upright images, while in Experiment 2, we used the difference in response between upright and inverted faces as another proxy for how face-like each category is according to the N170. In general, upside-down faces are known to be processed poorly compared to upright faces, while other object classes tend to be less affected by image inversion. This phenomenon is thus an additional means of characterizing whether or not the processing of a stimulus class is more or less “face-like.”

Briefly, we found in both experiments that robot faces occupy a middle ground between faces and non-faces. In terms of the N170, robot faces elicit an intermediate response that suggests they tend to share some critical features with human faces, but also lack some important image structure that the N170 is tuned to. We also find differential effects at the P100 and N170 that suggest that the status of robot faces as faces may vary depending on what stage of processing we consider. Specifically, robots’ substantial deviation from human-like appearance in local neighborhoods may have larger consequences at earlier stages of face processing, while their general adherence to the first-order geometry of human faces may lead them to be processed in a more face-like way at later stages of processing that entail holistic processing. We close by discussing the potential for more targeted analysis of critical image features that may lead robot faces to look more-or-less face-like, and the possibility that social interactions between robots and humans may affect the status of robot faces within the extended face network.

## Experiment 1: How do face-sensitive ERP components respond to robot faces?

In our first experiment, our goal was to compare the response of face-sensitive ERP components (the P100 and N170) to human faces, robot faces, and non-face objects. Specifically, we presented participants with photographs depicting human faces, computer-generated faces created from those photographs, images of doll faces, robot faces, and images of clocks. These stimuli were selected so that we could compare the ERP response to robot faces relative to human faces and non-face objects, as well as face images that are similar to human faces, but also depict inanimate objects.

## Methods

### Participants

Participants between the ages of 18–22 years old were recruited from the North Dakota State University (NDSU) Undergraduate Psychology Study Pool. Our final sample was composed of 24 participants (15 female) who self-reported normal or corrected-to-normal vision and most of which were right-handed (n = 2 with mixed left-handed responses), as assessed by the Edinburgh Handedness Inventory^23^. An additional 4 participants completed the task, but we were unable to use their data due to an error in event marking during continuous EEG recording. Prior to the beginning of the experiment, we obtained informed consent from all participants. All experimental procedures described here were reviewed and approved by the North Dakota State University Institutional Review Board and were carried out in accordance with the principles described in the Declaration of Helsinki.

### Stimuli

**Figure 1:**
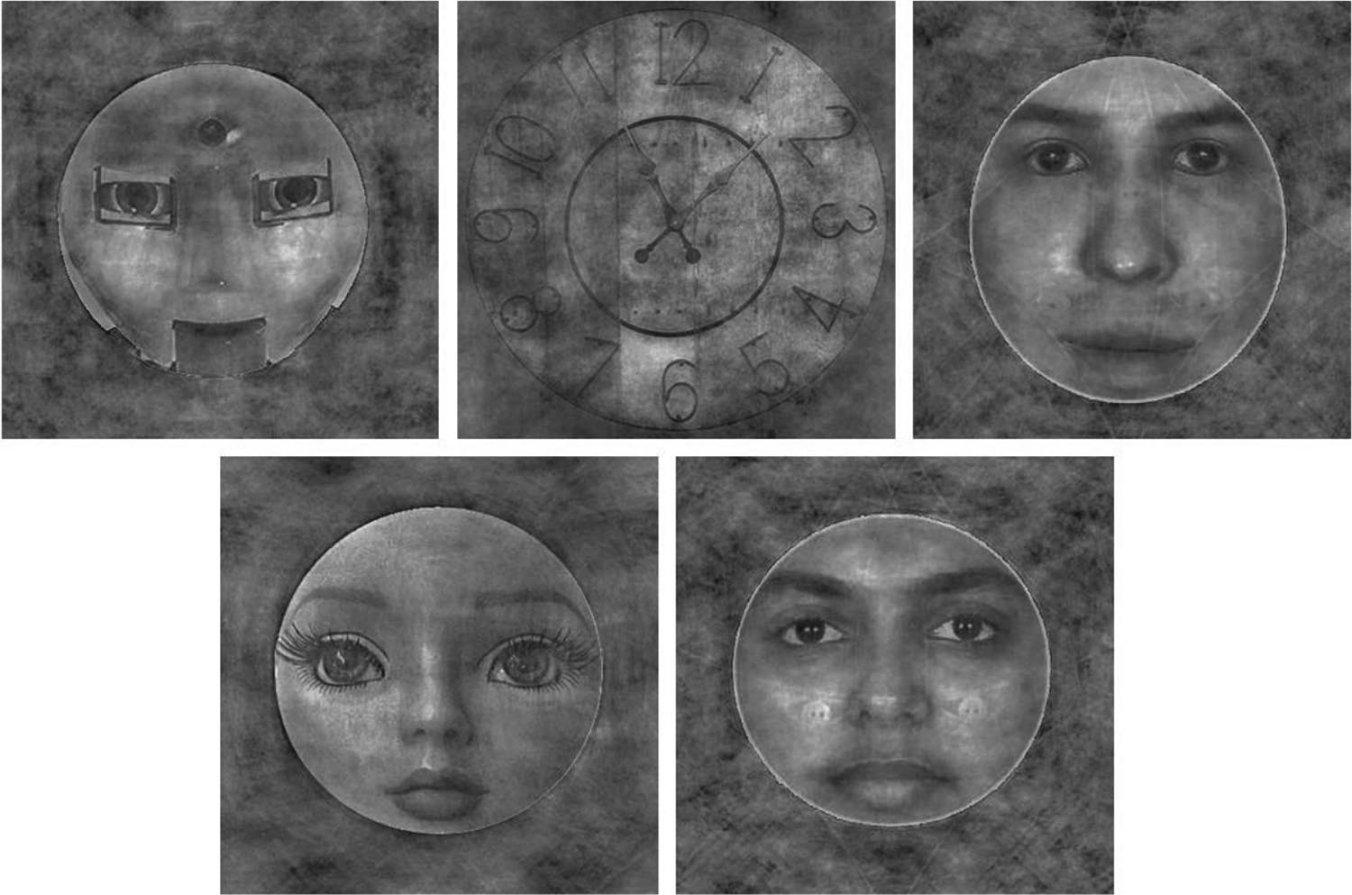
The images represent an example of each stimulus category following the application of power spectrum matching implemented using the MATLAB SHINE Toolbox (from left to right and top to bottom: robot, clock, CG, doll, and human face).

The doll, robot, and clock stimuli in this study were obtained from Google Image searches for those object categories, while photographic faces and their computer-generated (CG) counterparts were selected from a pre-existing database maintained by our laboratory. The computer-generated faces used here were created by importing our photographic face images into FaceGen, which is a commercial application for manipulating 3D face models using a morphable model of face appearance^24^. Briefly, the FaceGen program includes a model of both 3D shape and 2D surface appearance that is based on laser-scanned shape and surface data obtained from real people. The PhotoFit tool that we used allows users to import 2D images of a face, identify fiducial points in those images, and obtain a best-fitting 3D model of that image data that is manipulable in the FaceGen environment. These CG images thus strongly resemble the individuals in the photographs, but also look clearly computer-generated due to the limitations in the rendering of faces in the application^25^. Thirty images of each stimulus were included in our final task. We created these stimuli by converting all images greyscale and resizing them to 512 × 512 pixels. We then used the MATLAB SHINE Toolbox^26^ to equalize the power spectra of all stimulus images (Fig. 1), to minimize the possibility that any effects we observed at either target component were the result of category differences in low-level image properties.

An important limitation of our stimulus set is that we have not attempted to guarantee that there is equal variability across faces within our stimulus categories. This is a non-trivial problem in general and is important to acknowledge here given that robot faces in particular can vary substantially in appearance. By selecting images of social robots we have limited ourselves to robots that do have a face-like configuration of eyes, nose, and mouth (excluding robots that are only mechanical arms, for example, or that do not have an obvious face or head) but even within these constraints there can be variation in the materials, colors, and shapes used to design a robot. Given these limitations, our data should be interpreted with this in mind, though our use of power-spectrum and intensity histogram normalization allows us to make some precise statements about specific low-level features that are closely matched across all of our images. In particular, the intensity histogram (the distribution of grayscale values in our images) and the power spectrum of our images are closely matched within and across categories. This means that while there may be higher-order sources of variability that differ across our categories (perceived material properties or face configuration, for example) these specific low-level features are unlikely to underlie our results.

### Procedure

After obtaining consent, we measured the circumference of each participant's head to ensure the selection of a properly fitting Hydrocel Geodesic Sensor Net by EGI. Each 64-electrode net was soaked in a potassium chloride (KCl) solution for 5 min before being placed on the participant’s scalp. The net was then plugged into an EGI 400 NetAmps amplifier. Following, the impedance of each electrode was measured and those above 25 kΩ were adjusted and/or applied with more KCl solution until an adequate measurement was met.

The EEG recordings took place in an electrically-shielded and sound-attenuated chamber where our participants sat in front of a 1024 × 768 LCD monitor. EEG data was collected at a sampling rate of 250 Hz and NetStation v5.0 was used for the EEG recordings and event markings. Participants were presented with 30 unique images of each stimulus category. Each image was randomly presented twice in a pseudo-randomized order determined by functions in EPrime v2.0 during the session for a total of 300 images. The images were displayed on a white background for 500 ms with random interstimulus intervals (ISI) between 800 and 1500 ms. We asked participants to respond to these images by pressing a ‘person-like’ button for images of human, computer-generated, and doll faces, with one of their hands, and a ‘machine-like’ button for clock and robot faces with the other. We note that our use of this task does mean that robot faces and human faces should receive different responses, however the categories in question (human vs. machine) are orthogonal to the category of face, so we do not expect the assignment of different category labels to affect our target components. Goffaux et al.^27^ demonstrated that the overall nature of the categorization task assigned during ERP recording (gender categorization vs familiarity categorization) could affect the N170 amplitude’s sensitivity to faces filtered to remove high vs. low spatial frequency information, but this is distinct from a predicted effect of human vs. machine responses on either the P100 or N170. Instead, their result demonstrates that different categorization tasks may lead to differential reliance on specific aspects of face appearance, which in turn can affect the magnitude of the N170 in response to specific stimulus manipulations^28,29^. In our case, we chose a human/machine categorization task to require observers to attend to face images broadly in order to detect the multiple cues that may signal whether a face image depicts a human or human-like agent or a mechanical agent. Of course the different handedness of the two response categories is important to consider in this scenarion, and to account for the expected right-hand preference, the orientation of the response-box was turned 180 degrees after every participant, so that half of participants used their right hand for the ‘human” button, and the other half used their left. This task took about 15 min and the behavioral data was collected using EPrime v2.0.

## Results

### Behavioral data: human/machine classification

**Figure 2:**
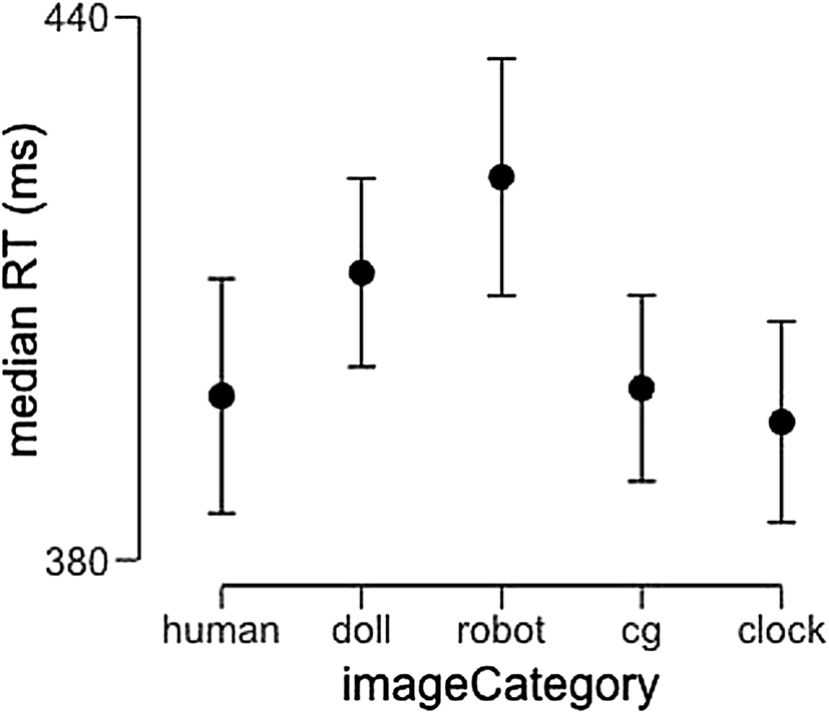
Average response latency for correct human/machine judgments across image categories. Error bars represent 95% confidence intervals.

For each participant, we calculated the median response time for correct human/machine judgments in each category. For all images of human faces, the correct answer was “human,” while robot faces and clocks should have been labeled “machine.” We analyzed these values using a 1-way repeated-measures ANOVA implemented in JASP^23^ with image category (real faces, CG faces, doll faces, robot faces, and clocks) as a within-subjects factor. This analysis revealed a significant main effect of image category (F(4,108) = 4.06, p = 0.004), and post-hoc testing revealed a significant pairwise difference between robot faces and clocks such that response latencies to robot faces were significantly slower (Mean diff. = 27.0 ms, s.e. = 8.08 ms, t = 3.35, p_bonf_ = 0.024). Pairwise differences between other conditions did not reach significance after correction for multiple comparisons. Average response latencies across participants are displayed in Fig. 2.

### ERP component analyses

We continued by examining both the mean amplitude and the peak latency of the P100 and N170 ERP components elicited by stimuli from each image category. For each participant, the continuous EEG recorded during the testing session was filtered using a 0.1–30 Hz bandpass filter, and then segmented into 1100 ms epochs using markers inserted into the EEG record during data acquisition. Each of these segments began 100 ms before stimulus onset and ended 1000 ms after stimulus onset. Each segment was baseline corrected by subtracting the average value calculated during the 100 ms baseline period from the entire waveform. Subsequently, we applied algorithms for identifying and removing eye movement artifacts from the data and used spherical spline interpolation to remove and replace any systematically bad channels in the sensor array. Finally, we calculated average ERPs for each participant by averaging together the data within each image category at each sensor, followed by the application of an average re-reference.

**Figure 3:**
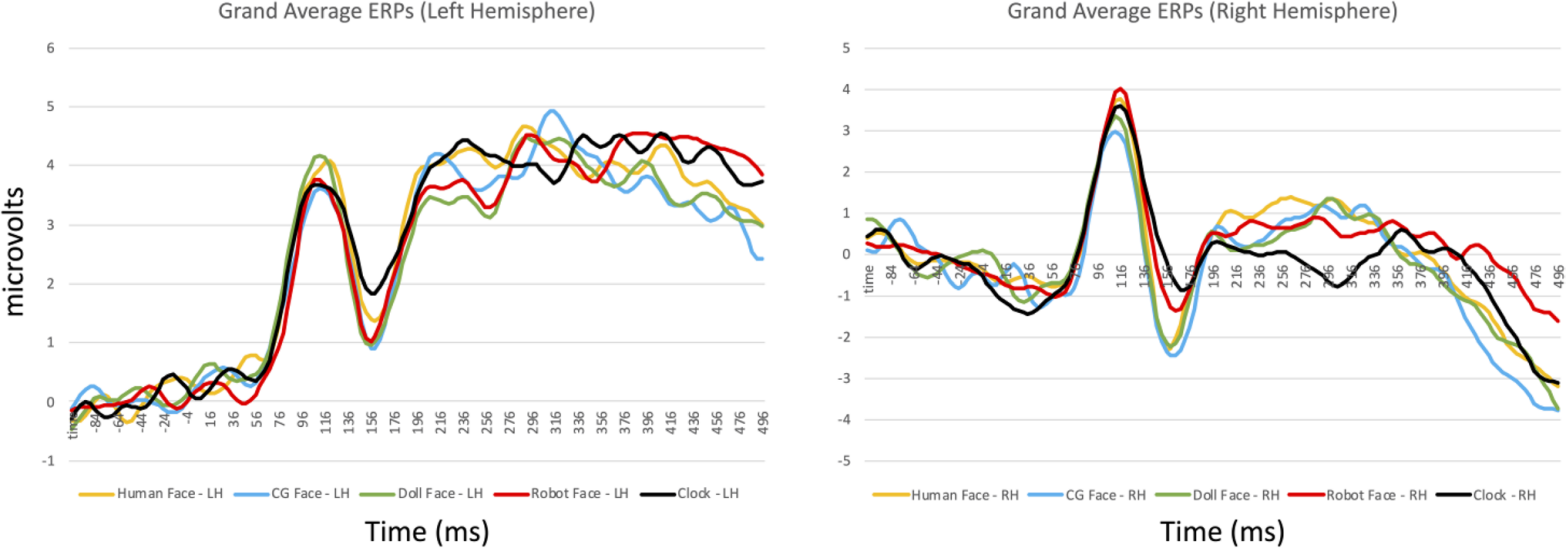
Grand average ERPs from the left (top) and right (bottom) hemispheres depicting the P100/N170 complex for all stimulus conditions in Experiment 1.

We identified time intervals and sensors of interest by visually inspecting a grand average ERP calculated across participants (Fig. 3), and locating maximal deflections corresponding to the components of interest. We analyzed both components using three sensors in the left hemisphere (sensors 29, 30, and 32 in the EGI sensor array) and three sensors in the right hemisphere (43, 44, and 47 in the EGI sensor array). These electrodes sites correspond closely to the T5/T6 locations in the 10–20 electrode system, which are positioned over occipito-temporal regions in the left and right hemisphere. The specific sensors we selected on the EGI sensor net are slightly more medial than T5 and T6, but are still positioned over the left and right occipito-temporal scalp. For a detailed analysis of the correspondence between EGI sensors and the 10–20 system, please see EGI’s technical paper on this topic, available at: https://www.egi.com/images/HydroCelGSN_10-10.pdf). We selected an interval of 92–136 ms to measure P100 amplitude and latency, and an interval of 140–192 ms to measure N170 amplitude and latency. For each ERP descriptor, we calculated these values for each participant and analyzed the resulting data using a 5 × 2 repeated measures ANOVA with image category (human face, doll face, cg face, robot face, and clock) and hemisphere (left or right) as within-subjects factors.

### P100 amplitude

Our analysis of the P100 mean amplitude yielded neither a significant main effect of image category (F(4,92) = 1.56, p = 0.19, partial η^2^ = 0.064) nor a main effect of hemisphere (F(1,23) = 2.06, p = 0.16, partial η^2^ = 0.082). The interaction between these two factors also did not reach significance (F(4,92) = 1.61, p = 0.18, partial η^2^ = 0.065).

### P100 latency

**Figure 4:**
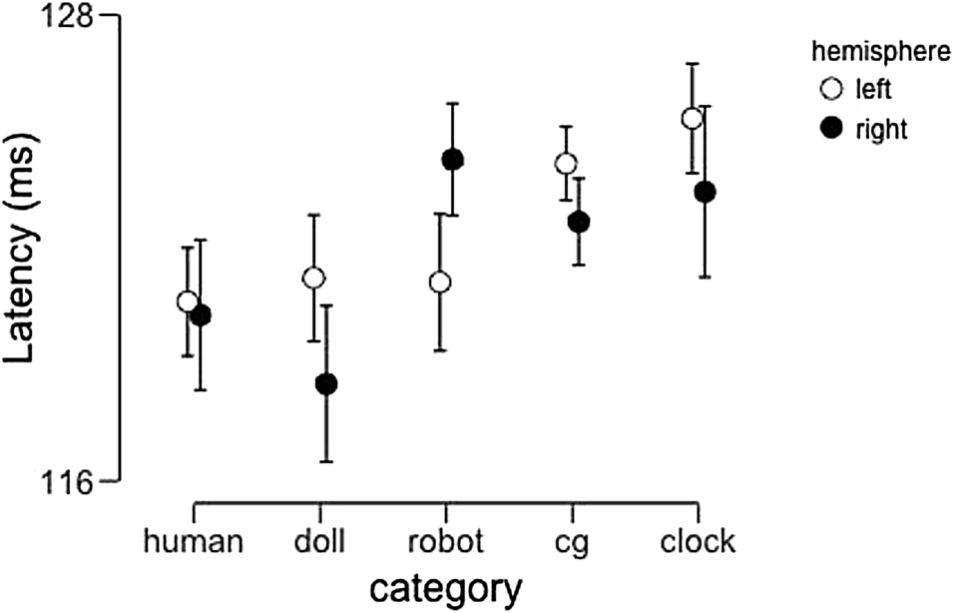
Average P100 peak latencies across participants as a function of image category and hemisphere. Error bars represent 95% confidence intervals.

Our analysis of the P100 latency-to-peak data (Fig. 4) yielded a significant main effect of image category (F(4,92) = 2.86, p = 0.028, partial η^2^ = 0.11). In subsequent post-hoc tests, no pairwise difference between categories reached significance following corrections for multiple comparisons Neither the main effect of hemisphere (F(1,23) = 0.18, p = 0.68, partial η^2^ = 0.008) nor the interaction between these two factors (F(4,92) = 1.48, p = 0.22, partial η^2^ = 0.060) reached significance.

### N170 amplitude

**Figure 5:**
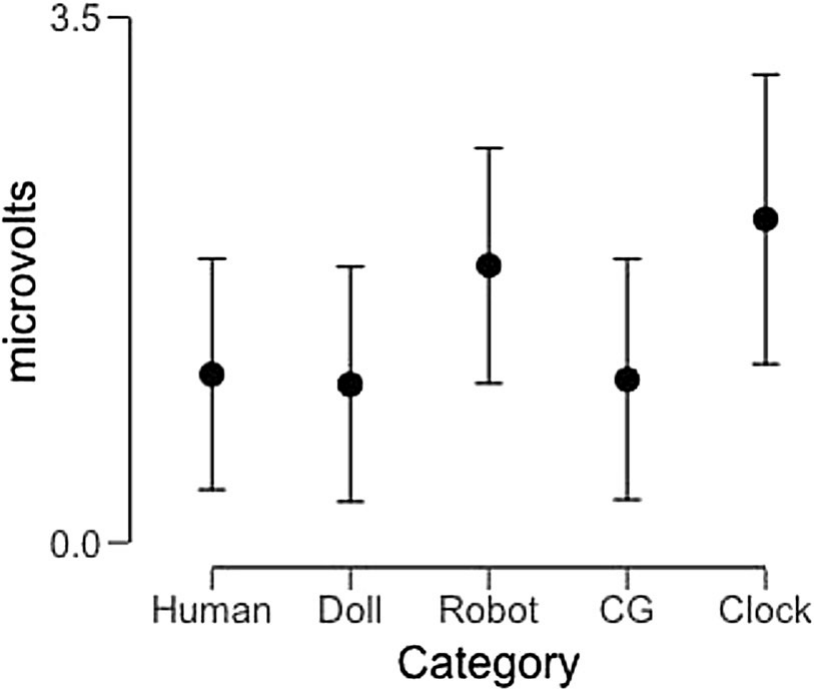
Average N170 mean amplitude across participants as a function of image category (collapsed across hemisphere). Error bars represent 95% confidence intervals.

This analysis revealed a main effect of image category (F(4,92) = 2.85, p = 0.028, partial η^2^ = 0.11), but neither a main effect of hemisphere (F(1,23) = 2.76, p = 0.11, partial η^2^ = 0.11) nor a significant interaction between these factors (F(4,92) = 1.38, p = 0.25, partial η^2^ = 0.056). Post-hoc tests revealed that the main effect of image category was the result of significant pairwise differences between robot faces and CG faces (t = 3.34, p_bonf_ = 0.016, Cohen’s d = 0.68) while pairwise differences between human and robot faces (t = 2.87, p_bonf_ = 0.06, Cohen’s d = 0.59) and between doll faces and robot faces (t = 2.99, p_bonf_ = 0.057, Cohen’s d = 0.59) did not reach significance. In Fig. 5 we display average mean amplitude across participants as a function of image category.

### N170 latency

**Figure 6:**
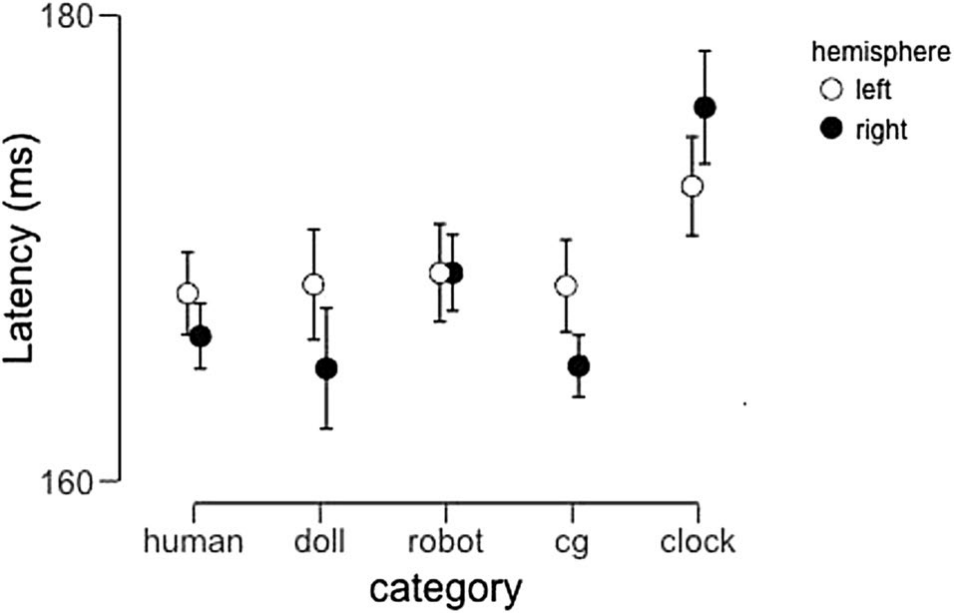
Average N170 Latency across participants as a function of image category and hemisphere. Error bars represent 95% confidence intervals.

This analysis revealed a significant main effect of image category (F(4,92) = 4.48, p = 0.002, partial η^2^ = 0.16), but neither a main effect of hemisphere (F(1,23) = 0.36, p = 0.55, partial η^2^ = 0.016) nor an interaction between these factors (F(4,92) = 1.88, p = 0.12, partial η^2^ = 0.076). Post-hoc tests revealed that the main effect of image category was the result of pairwise differences between human faces and clocks (t = − 3.63, p_bonf_ = 0.007, Cohen’s d = 0.74), doll faces and clocks (t = − 3.07, p_bonf_ = 0.035, Cohen’s d = 0.63), and CG faces and clocks (t = − 4.43, p_bonf_ < 0.001, Cohen’s d = 0.90). Critically, the difference between robot faces and clocks did not reach significance (t = 2.76, p_bonf_ = 0.083, Cohen’s d = 0.56). In Fig. 6, we display the average N170 latency across participants as a function of image category and hemisphere.

## Discussion

The results of our first experiment provide initial evidence that robot faces maybe processed as intermediate stimuli that are neither purely face-like nor purely object-like, while artificial human faces elicit responses that are decidedly face-like. This latter feature of our data is consistent with previous reports indicating limited effects of artificial appearance on the N170 amplitude and latency^30,31^. There are substantial differences in appearance between real human faces, dolls, and computer-generated faces, and artificial appearance is known to disrupt performance in a range of face recognition tasks. Artificial faces are harder to remember, for example^32^, and elicit different social evaluations of qualities like trustworthiness even when identity is matched between real and artificial images^33^. Despite the existence of a perceptual category boundary between real and artificial faces^34^ and the various recognition deficits that appear to accompany artificial face appearance, the neural processing of these images at the N170 is similar to that of real human faces. This is an important observation because it demonstrates that the tuning of the N170 to face appearance is broad enough to include these categories, and also because it suggests that strong attribution of a mind, intentions, or other dimensions of social agency (which are diminished for artificial faces^33^) are not necessary to elicit a robust N170 response.

Compared to these artificial human face conditions, the differences between our robot condition and human faces are more complex. While the profound differences in facial appearance between these categories did not elicit differential processing at the P100, a complicated picture emerges from the N170’s response properties. The amplitude data suggests at best only weak differentiation of robot faces from human faces, and no evidence of a difference between robots and clocks. This outcome could be interpreted as support for the hypothesis that robots are processed in an intermediate fashion, but this reasoning relies heavily on null results rather than statistically meaningful differences between conditions and is thus suspect. However, the N170 latency data is somewhat clearer in that it reveals that human-like faces of all types differ from non-face objects, implying strong differentiation of human-like faces from objects. By comparison, robot faces do not clearly from clocks, nor do they reliably differ from human faces. Again, this is somewhat difficult data to interpret as the response to robots is again not statistically different from either of the two extremes (real human faces and clocks), while other artificial faces (CG faces and dolls) do differ from the non-face response clearly. This is of course a “difference of significances” scenario rather than the observation of a significant difference between critical categories, making it challenging to draw a clear conclusion about the status of robot faces relative to human faces and clocks. Robot faces could be processed in a manner that is neither entirely face-like, nor entirely object-like, but we should be cautious with this interpretation based solely on these data.

Also, our first experiment does not allow us to rule out an important alternative account. Specifically, the amount of variability across human faces, robot faces, and clocks was not explicitly measured or controlled in our stimulus set, and any differences in within-category image variability could contribute to the results we observed. In particular, though we did match low-level stimulus properties including the intensity histogram and the power spectrum across images, higher-level aspects of appearance including perceived material properties and inferred shape or curvature may vary by different amounts within these categories. Inter-stimulus perceptual variance (ISPV) has been shown to modulate N170 amplitudes in particular^35^, with larger ISPV values associated with smaller N170 amplitudes due to increased trial-by-trial jitter of the N170. Our results are therefore consistent with a stimulus set that has increased ISPV for robot faces compared to human faces. This could be the case in our stimulus set; robot faces vary substantially across manufacturers according to the roles they are intended to occupy, while our CG faces were matched to the identities of our real human faces. The intermediate status of robot faces could thus reflect an artifact of our stimulus set rather than the ambiguous category status of robots.

To help address this issue and further examine the extent to which face-specific processing includes robot faces, in our second experiment we chose to investigate the face inversion effect (FIE) at our target components for human faces, robot faces, and clocks. In terms of ERP responses, the FIE refers to the increased amplitude and latency of the N170 component in response to inverted face images^36^. We suggest that this is a useful way to address the issue of potential category differences in ISPV because the effect of inversion on amplitude follows from an ISPV-based account only if we assume that inversion increases stimulus homogeneity, which is itself an indicator of orientation-dependent processing. Further, latency effects on the N170 with inversion are to our knowledge not easily explained in an ISPV account^37^, making these markers of the FIE a more robust indicator of face-specific processing for a particular stimulus category. We hypothesized that if robot faces are indeed a boundary category that lies between faces and non-faces, we may see an FIE that is intermediate to that observed for human faces and clocks. Alternatively, if the FIE for robot faces clearly mirrors what we observe in either comparison category, this may make a stronger case for robots being considered unambiguously as either faces or non-faces at these stages of visual processing.

## Experiment 2: Do robot faces yield a face inversion effect at face-sensitive ERP components?

In Experiment 2, we investigated the P100 and N170 responses to upright and inverted human, robot, and clock faces. Both components have been reported to exhibit a face inversion effect (FIE) such that inverted faces lead to larger amplitudes, and our goal was to use this effect as a proxy for face-like processing.

## Methods

### Participants

Our final sample was composed of 24 students in the NDSU Undergraduate Psychology Study Pool (Female = 16) between the ages of 18–23. Only 1 participant reported mixed left-handedness and all participants self-reported normal or corrected-to-normal vision. We obtained informed consent from all participants prior to the beginning of the experiment.

### Stimuli

**Figure 7:**
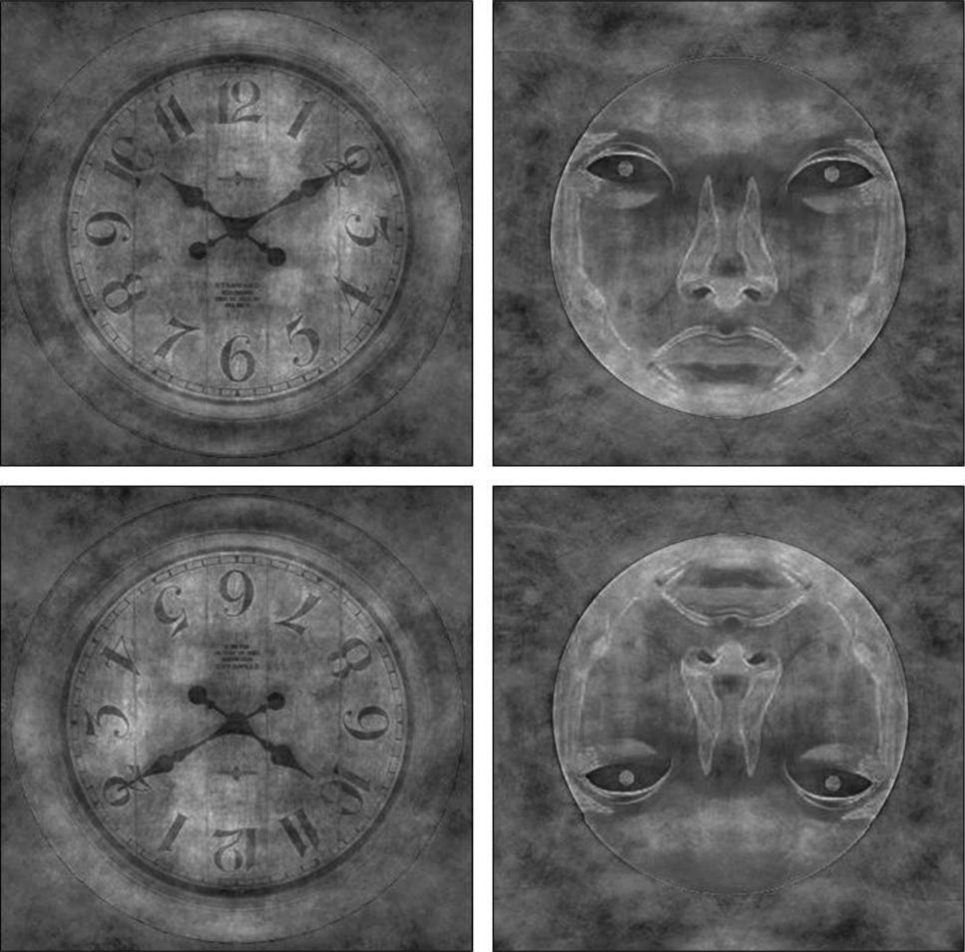
Example images from each stimulus category presented in upright and inverted orientations.

For this experiment, we presented participants with right-side-up and inverted clock, robot, and human face images (Fig. 7). Because the ERP components of doll and computer-generated facial stimuli were so similar to that of human face images in experiment 1, we excluded them from this study. The images for this study were taken from experiment 1 and therefore, did not undergo editing. However, many robot face images looked similar when inverted, so we handpicked robot face images that looked noticeably inverted and ended up with 18 images. We then randomly chose 18 images from the other stimulus categories. These 54 images were duplicated and inverted, leaving us with 54 upright and 54 inverted stimuli.

### Procedure

All recording software and procedures for preparing the participant and the Hydrocel Geodesic Sensor Net for the session were identical to those reported in Experiment 1.

Participants were presented with each stimulus in a pseudo-randomized order four times for a total of 432 trials. The images were displayed for 500 ms on a white background with random ISIs ranging between 800 and 1500 ms. Participants were asked to respond to the images by pressing a ‘human’ button for upright and inverted human faces and a ‘machine” button for upright and inverted clock and robot face images. Participants were asked to use one hand for each button. The response box was flipped 180° for half of our participants to balance which hand corresponded to which response cross participants.

## Results

### Behavioral data: human/machine classification

For each participant, we calculated the median response time for correct human/machine judgments in each category. For all images of human faces, the correct answer was “human,” while robot faces and clocks should have been labeled “machine.” We analyzed these values using a 2-way repeated-measures ANOVA implemented in JASP^27^ with image category (real faces, robot faces, and clocks) and orientation (upright and inverted) as within-subjects factors. The main effect of image category (F(2,44) = 2.85, p = 0.069) did not reach significance, nor did the main effect of orientation (F(1,22) = 1.67, p = 0.21) or the interaction between these factors (F(2,44) = 0.80, p = 0.46).

### ERP component analyses

**Figure 8:**
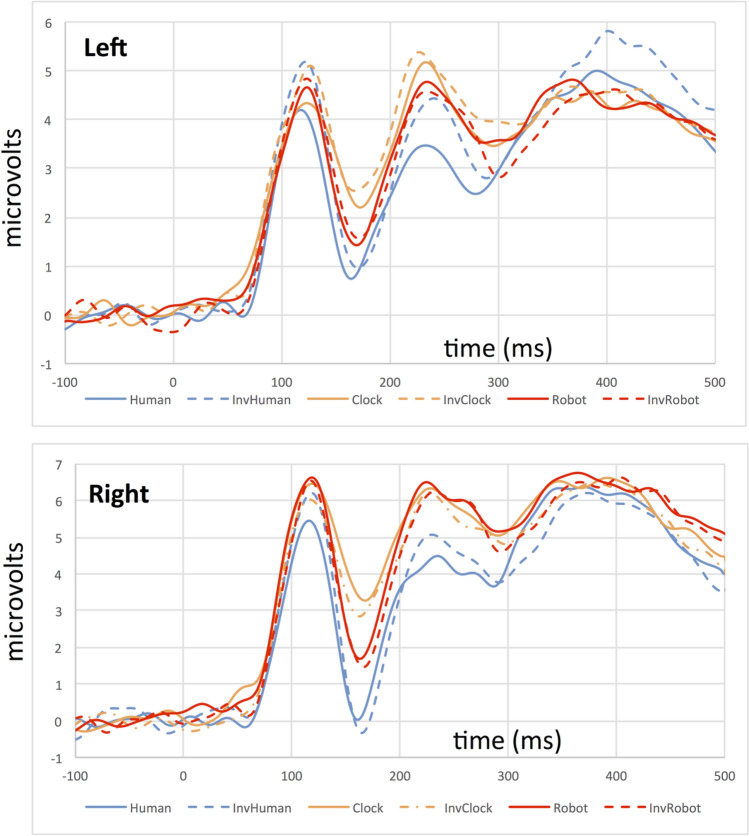
Grand average ERP waveforms for the left (top) and right (bottom) hemisphere depicting the P100/N170 complex for all conditions in Experiment 2. Solid lines depict the responses to upright stimuli, while dashed lines depict responses to inverted stimuli.

As in Experiment 1, we identified sensors and time windows of interest by inspecting the grand average ERP calculated across participants (Fig. 8). We analyzed the P100 and N170 components at the same sensors as in Experiment 1, and used a time window of 84–124 ms to measure the P100 amplitude and latency, and a time window of 148–212 ms to measure the N170 amplitude and latency.

### P100 amplitude

This analysis revealed a significant main effect of image category (F(2,44) = 5.32, p = 0.009, partial η^2^ = 0.195), but no main effects of orientation (F(1,22) = 2.10, p = 0.16, partial η^2^ = 0.087) or hemisphere (F(1,22) = 3.22, p = 0.086, partial η^2^ = 0.13). We also observed significant interactions between image category and orientation (F(2,44) = 14.7, p < 0.001, partial η^2^ = 0.41) and between orientation and hemisphere (F(1,22) = 8.35, p = 0.009, partial η^2^ = 0.275). No other interactions reached significance.

**Figure 9:**
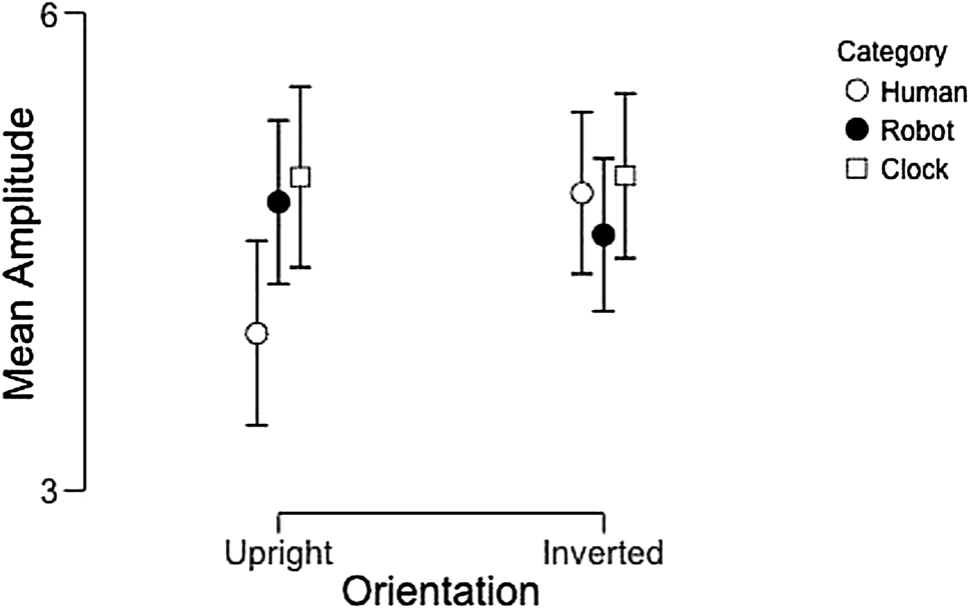
Average P100 amplitude across participants as a function of image category and orientation (collapsing across left/right hemisphere). Error bars represent 95% confidence intervals.

Post-hoc testing revealed that the main effect of image category was the result of a significant pairwise difference between responses to human faces and clocks (t = − 3.84, p < 0.001, Cohen’s d = 0.81) The interaction between image category and orientation was the result of a significant orientation effect for human faces such that inverted faces elicited a more positive P100 peak than upright faces, but no such difference between upright and oriented clocks or robot faces (see Fig. 9).

### P100 latency

We observed no significant main effects or interactions on the P100 latency to peak. Neither image category, orientation, or hemisphere affected this aspect of participants’ waveforms.

### N170 amplitude

This analysis revealed a main effect of image category (F(2,44) = 40.2, p < 0.001, partial η^2^ = 0.65), but no main effects of orientation (F(1,22) = 0.55, p = 0.41, partial η^2^ = 0.024) or hemisphere (F(1,22) = 1.54, p = 0.23, partial η^2^ = 0.065). The lack of a main effect of orientation is surprising given prior reports of inverted faces eliciting more negative N170 amplitudes than upright faces, but we did also observe a significant interaction between orientation and hemisphere (F(1,22) = 4.44, p = 0.047, partial η^2^ = 0.17). No other interactions reached significance.

**Figure 10:**
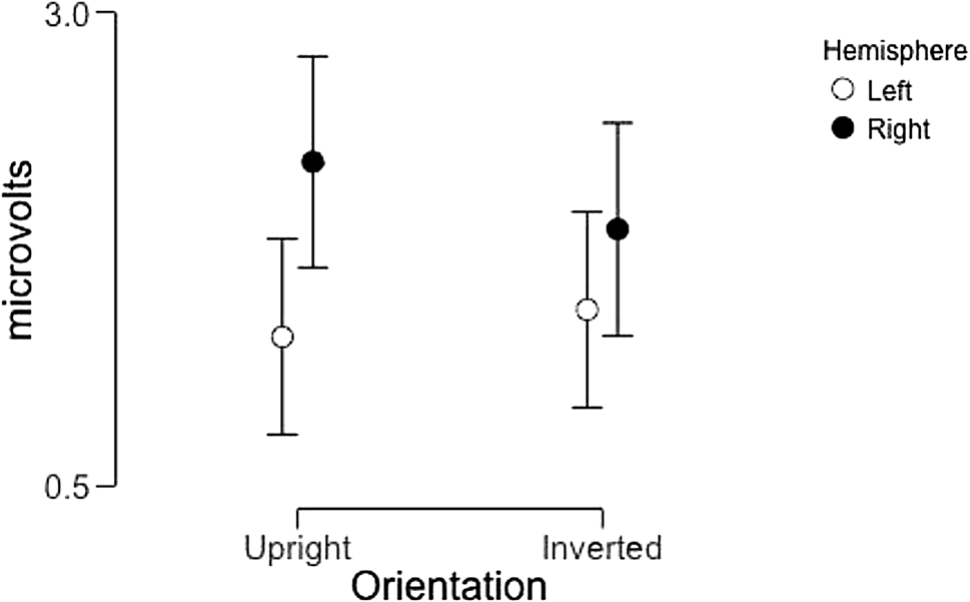
Average N170 amplitude across participants as a function of image orientation and hemisphere (collapsed across image category). Error bars represent 95% confidence intervals.

The main effect of image category was driven by significant pairwise differences between all three image categories (t > 6.11 in all cases, p < 0.001 for all, Cohen’s d > 1.25 in all cases). The interaction between orientation and hemisphere was driven by a larger difference between left and right hemisphere responses for upright stimuli compared to inverted stimuli (Fig. 10). This is to some extent consistent with a face inversion effect in the predicted direction that is more evident in the right hemisphere than the left, but we note that the pairwise difference between upright and inverted amplitudes in the right hemisphere did not reach significance.

### N170 latency

**Figure 11:**
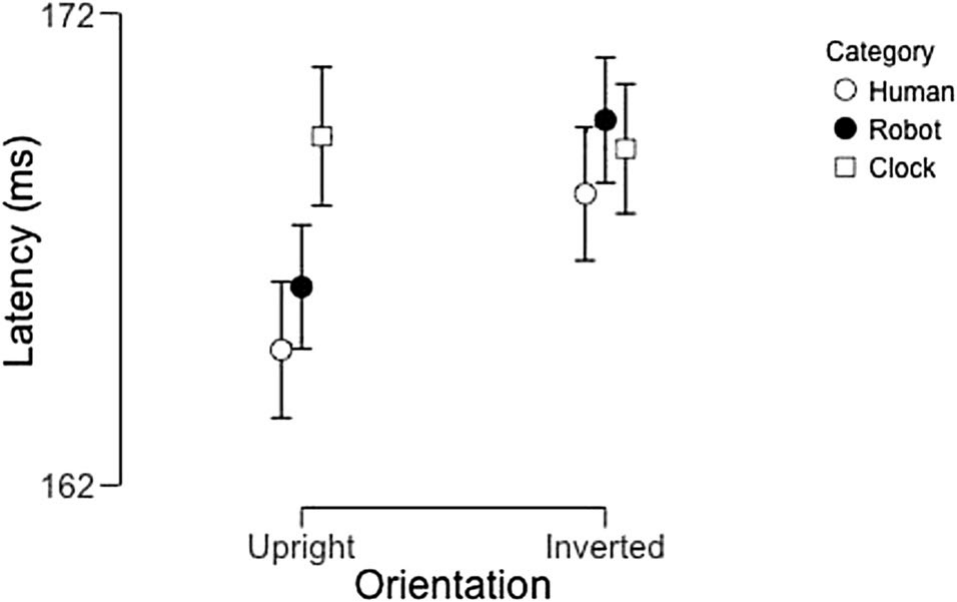
Average N170 latencies across participants as a function of image category and orientation. Error bars represent 95% confidence intervals.

This analysis revealed only a significant interaction between image category and orientation (F(2,44) = 3.2, p = 0.05, partial η^2^ = 0.13). No other main effects of interactions reached significance. Post-hoc testing revealed that this interaction was driven by significant inversion effects for human and robot faces such that inverted faces elicited slower latencies to peak than upright faces, but there was no effect of image orientation on clock images (Fig. 11).

### General discussion

Across two experiments, we have found that the neural response to robot faces has properties that suggest robot faces indeed lie on the boundary between faces and non-face objects. In our first experiment, we found that the N170 amplitude elicited by robot faces was intermediate to the response to human faces of multiple types and non-face objects. In Experiment 2, we further demonstrated that the face inversion effect was generally not evident for non-face objects, but was evident to a limited extent for robot faces. To the extent that we should accept the inversion effect as a proxy for the engagement of face-specific processing, these results together suggest that robot faces are capable of engaging these mechanisms to a limited degree while still different enough from human faces to elicit a different response.

In particular, in Experiment 2 we found that the face inversion effect was not evident for robot faces at the P100, but was observed in the N170 latency data. It is overall somewhat surprising, however, that we did not observe an enhanced N170 amplitude to inverted face images generally, as reported in Ref.^10^. It is difficult for us to account for the absence of an inversion effect on amplitude in this case, as well as other features of our data, like the absence of a clear P200 component in Experiment 2. In both cases, we can only speculate that either some property of these stimuli (for example the manner in which we cropped and normalized our images) or properties of the stimuli considered as a whole (variability across images within a category, e.g.) may have reduced the size of this effect in our data. Regardless, we are still able to discuss the impact of inversion on the response to human and robot faces via the latency effects that we did observe. Despite the fact that the P100 generally reflects low-level processing that can be affected by basic image properties, this component also demonstrates some sensitivity to upright vs. inverted face images^9^. However, in terms of the extended face network in extrastriate cortex, this early component also appears to reflect a different stage of processing than the subsequent N170^38^. In particular, the P100 has been associated with part-based, as opposed to holistic, face processing^5^, but this interpretation is somewhat at odds with theoretical accounts of face inversion as an index of holistic processing. For our purposes, we think that the presence of a robot face inversion effect at the N170 but not at the P100 reveals an interesting functional difference between robot and human faces regardless of the precise mechanistic differences between the processes indexed by these two components. Whatever processes these two components reflect, there is some property of robot faces that passes muster with the later component but is not sufficient to elicit face-specific processing from the P100.

One intriguing possibility is that social categorization may play an important role in determining how the processes indexed by these two components engage with robot faces versus human faces. In terms of behavioral responses, there is some evidence that out-group faces are less susceptible than in-group faces to manipulations that affect holistic processing^39^, including the face inversion effect^40^. Neural responses also appear to be sensitive to social categories as well: Colombatto and McCarthy^9^ reported that the face inversion effect at the P100 was only evident for White faces viewed by White observers, and not evident for Black faces. This is in contrast to prior reports that demonstrated sensitivity to face orientation for White and Black faces at the N170^41^, which suggests that these two components differ in part based on the extent to which social information modulates the nature of the neural response. We therefore suggest that one account of our data is that the social other-ness of robots is sufficient to exclude them from face-specific processing at an early cortical stage, but does not exclude them from subsequent face-specific processing at the N170. Robots are indeed subject to social categorization effects that affect how human observers interact with them. Eyssel and Kuchenbrandt^42^ demonstrated that robots can be favoured as in-group or out-group members based solely on the name/label they are given without further interaction. This suggests that robots, though they are objects, not only act as social beings but may also be assigned to social categories in the same way as human faces. One potentially useful follow-up to the current experiments would be an examination of the strength of the other-race effect for robot faces (defined by perceptual discrimination abilities or memory biases) compared to human faces in relation to the neural effects we have reported here. Indeed, relationships between specific neural responses and the eeriness of artificial faces that lie in the so-called “Uncanny Valley” suggest that in this important limiting case, the other-ness of some artificial agents is sufficient to elicit differential processing in the ventromedial prefrontal cortex^43^. There are important methodological issues to consider in carrying out such a study, particularly the challenge of ensuring that inter-item variability is comparable within robot and human face categories. If robot faces were simply more distinct from one another at an image level than human faces, one might observe a difference between conditions that has nothing to do with in-group/out-group categorization of face images. This is a tractable problem, however, which could be addressed via independent characterization of inter-item similarity between candidate human and robot faces. Developing a stimulus set with accompanying metadata along these lines could be a highly useful contribution to the emerging study of non-human social agents in perceptual and cognitive tasks. Along similar lines, creating morphed face images depicting a blend of human and robot appearance could also be a useful way to titrate the amount of robotic appearance and control variability. Such morphs would be challenging to create given the difference in material appearance (skin vs. metal or plastic) between humans and robots as well as the difference in local feature shape, but could be a useful tool for closely examining the boundary between robot faces and human faces.

Another interesting way to examine the role of social categorization in affecting the inclusion or exclusion of robot faces from face-specific processing is to manipulate how robots are socially categorized through experience and exposure. Social categorization and social identity have both been reported to affect ERP indices of face processing when human faces are presented to observers^44,45^ so it is perhaps not too difficult to imagine that these factors could also affect the neural response to robot faces. Several behavioral results demonstrate that brief social interactions can lead to changes in how human observers evaluate robot agents. A social exchange between humans and robots can change beliefs and actions towards robots^46^. People can feel empathy towards a robot from simply hearing a story about it, and furthermore, this empathetic feeling makes participants hesitate when told to strike it^47^. Social exchanges between robots and children have also been shown to change more complex evaluations regarding robots. For example, in Shen^47^, following a 15-min interaction with a robot named ‘Robovie,” children developed the idea that Robovie had feelings, interests, and was intelligent, but like objects, Robovie did not have civil liberties. Of particular relevance, results from an ERP study conducted by Chammat et al.^48^ suggested that emotional robot faces elicit similar ERP responses to emotionally charged human faces, even when using a non-humanoid robot. These results highlight how context and experience can affect the way people attribute social and personality characteristics to robots. If social and personality characteristics affect the manner in which human faces are processed by the visual system, perhaps these same characteristics (and changes in how people evaluate them) could also modulate the neural response to robot faces. This is of course pure speculation on our part (and we emphasize is not in any way implied by our current results), but we raise this issue to highlight the potential for continued study of the visual processing of robot faces to advance our understanding of how perceptual and social aspects of face stimuli may both contribute to the processing of face images at different stages of the visual system.

Overall, our data demonstrate how the tuning of neural mechanisms for face recognition is complex. Robot faces are a useful stimulus class for examining a wide range of perceptual and social factors that affect face processing, largely because of their residence at the border between face and object. As robotic agents become more common in a range of everyday settings, understanding the nature of this boundary and what factors can affect its topography will be increasingly important.

## Data Availability

The materials used to run this study and the data files supporting the analyses reported here are available via the Open Science Framework at the following link: https://osf.io/br6zx/.

## References

[1] Pitcher D, Walsh V, Yovel G, Duchaine B (2007). TMS evidence for the involvement of the right occipital face area in early face processing. Curr. Biol..

[2] Kanwisher N, Yovel G (2006). The fusiform face area: A cortical region specialized for the perception of faces. Philos. Trans. R. Soc. Lond. Ser. B Biol. Sci..

[3] Schobert AK, Corradi-Dell'Acqua C, Frühholz S, van der Zwaag W, Vuilleumier P (2018). Functional organization of face processing in the human superior temporal sulcus: A 7T high-resolution fMRI study. Soc. Cogn. Affect. Neurosci..

[4] Wang X, Zhen Z, Song Y, Huang L, Kong X, Liu J (2016). The hierarchical structure of the face network revealed by its functional connectivity pattern. J. Neurosci..

[5] Pitcher D, Duchaine B, Walsh V, Yovel G, Kanwisher N (2011). The role of lateral occipital face and object areas in the face inversion effect. Neuropsychologia.

[6] Zhang J, Li X, Song Y, Liu J (2012). The fusiform face area is engaged in holistic, not parts-based, representation of faces. PLoS One.

[7] Deen B, Koldewyn K, Kanwisher N, Saxe R (2015). Functional organization of social perception and cognition in the superior temporal sulcus. Cereb. Cortex..

[8] Herrmann MJ, Ehlis AC, Ellgring H, Fallgatter AJ (2005). Early stages (P100) of face perception in humans as measured with event-related potentials (ERPs). J. Neural Transm..

[9] Colombatto C, McCarthy G (2017). The effects of face inversion and face race on the P100 ERP. J. Cogn. Neurosci..

[10] Rossion B, Gauthier I, Tarr MJ (2000). The N170 occipito-temporal component is delayed and enhanced to inverted faces but not to inverted objects: An electrophysiological account of face-specific processes in the human brain. NeuroReport.

[11] Bentin S, McCarthy G, Perez E, Puce A, Allison T (1996). Electrophysiological studies of face perception in humans. J. Cogn. Neurosci..

[12] Itier RJ, Alain C, Sedore K, McIntosh AR (2007). Early face processing specificity: It's in the eyes!. J. Cogn. Neurosci..

[13] Gandhi T, Suresh N, Sinha P (2012). EEG responses to facial-contrast chimeras. J. Integr. Neurosci..

[14] Omer Y, Sapir R, Hatuka Y, Yovel G (2019). What is a face? Critical features for face detection. Perception.

[15] Paras CL, Webster MA (2013). Stimulus requirements for face perception: An analysis based on "totem poles". Front. Psychol..

[16] Wardle, S. G., Seymour, K. & Taubert, J. Characterizing the response to face pareidolia in human category-selective visual cortex. *bioRxiv.* 233387 (2017).

[17] Moulson MC, Balas B, Nelson C, Sinha P (2011). EEG correlates of categorical and graded face perception. Neuropsychologia.

[18] Meng M, Cherian T, Singal G, Sinha P (2012). Lateralization of face processing in the human brain. Proc. R. Soc. B..

[19] Ghazali AS, Ham J, Barakova EI, Markpoulos P (2018). Effects of robot facial characteristics and gender in persuasive human–robot interaction. Front. Robot. AI.

[20] Broadbent E, Kmart V, Li X, Sollers J, Stamford RQ, MacDonald BA, Werner DM (2013). Robots with display screens: A robot with a more humanlike face display is perceived to have more mind and a better personality. PLoS One.

[21] Arita A, Hiraki K, Kanda T, Ishiguro H (2004). Can we talk to robots? Ten-month-old infants expected interactive humanoid robots to be talked to by persons. Cognition.

[22] Mathur MB, Reichling DB (2015). Navigating a social world with robot partners: A quantitative cartography of the uncanny valley. Cognition.

[23] Oldfield RC (1971). The assessment and analysis of handedness: The edinburgh inventory. Neuropsycholgia.

[24] Blanz, V. & Vetter, T. A morphable model for the synthesis of 3D faces. In *Proceedings of the 26th Annual Conference on Computer Graphics and Interactive Techniques, July 1999,* 187–194 (1999).

[25] Balas B, Horski J (2012). You can take the eyes out of the doll, but. Perception.

[26] Willenbockel V, Sadr J, Fiset D, Horne GO, Gosselin F, Tanaka JW (2010). Controlling low-level image properties: The SHINE toolbox. Behav. Res. Methods..

[27] JASP Team. JASP (Version 0.8.5.1)[Computer software] (2018).

[28] Goffaux V, Jemel B, Jacques C, Rossion B, Schyns PG (2003). ERP evidence for task modulations on face perceptual processing at different spatial scales. Cogn. Sci..

[29] Senholzi KB, Ito TA (2013). Structural face encoding: How task affects the N170's sensitivity to race. Soc. Cogn. Affect. Neurosci..

[30] Balas B, Koldewyn K (2013). Early visual ERP sensitivity to the species and animacy of faces. Neuropsychologia.

[31] Wheatley T, Weinberg A, Looser C, Moran T, Hajcak G (2011). Mind perception: Real but not artificial faces sustain neural activity beyond the N170/VPP. PLoS One..

[32] Balas B, Pacella J (2015). Artificial faces are harder to remember. Comput. Hum. Behav..

[33] Balas B, Pacella J (2017). Trustworthiness perception is disrupted in artificial faces. Comput. Hum. Behav..

[34] Looser CE, Wheatley T (2010). The tipping point of animacy. How, when, and where we perceive life in a face. Psychol. Sci..

[35] Thierry G, Martin CD, Downing P, Pegna AJ (2007). Controlling for interstimulus perceptual variance abolishes N170 face selectivity. Nat. Neurosci..

[36] Rossion B, Jacques C (2008). Does physical interstimulus variance account for early electrophysiological face sensitive responses in the human brain? Ten lessons on the N170. Neuroimage.

[37] Desjardins JA, Segalowitz SJ (2013). Deconstructing the early visual electrocortical responses to face and house stimuli. J. Vis..

[38] Michel C, Caldara R, Rossion B (2006). Same-race faces are processed more holistically than other-race faces. Vis. Cogn..

[39] Wiese H (2013). Do neural correlates of face expertise vary with task demands? Event-related potential correlates of own- and other-race face inversion. Front. Hum. Neurosci..

[40] Balas B, Nelson CA (2010). The role of face shape and pigmentation in other-race face perception: An electrophysiological study. Neuropsychologia.

[41] Eyssel F, Kuchenbrandt D (2012). Social categorization of social robots: Anthropomorphism as a function of robot group membership. Br. J. Soc. Psychol..

[42] Horstmann AC, Bock N, Linhuber E, Szczuka JM, Straβmann C, Krämer NC (2018). Do a robot’s social skills and its objection discourage interactants from switching the robot off?. PLoS One.

[43] Rosenthal-von der Pütten AM, Krämer NC, Maderwald S, Brand M, Grabenhorst F (2019). Neural mechanisms for accepting and rejecting artificial social partners in the uncanny valley. J. Neurosci..

[44] Darling, K., Nandy, P. & Breazeal, C. Empathic Concern and the Effect of stories in human–robot interaction. In *24th IEEE International Symposium on Robot and Human Interactive Communication (RO-MAN)*, 770–775 (2015).

[45] Derks B, Stedehouder J, Ito TA (2015). Social identity modifies face perception: An ERP study of social categorization. Soc. Cogn. Affect. Neurosci..

[46] Rollins L, Olsen A, Evans M (2020). Social categorization modulates own-age bias in face recognition and ERP correlates of face processing. Neuropsychologia.

[47] Shen S (2012). “Robovie, you’ll have to go into the closet now:” Children’s social and moral relationships with a humanoid robot. Dev. Psychol..

[48] Chammat M, Foucher A, Nadel J, Duba S (2010). Reading sadness beyond human faces. Brain Res..

